# *Dirofilaria asiatica* Infection and Asymptomatic Microfilaremia, Sri Lanka, 2025

**DOI:** 10.3201/eid3211.260015

**Published:** 2026-11

**Authors:** Dilini Mataraarachchi, Iroshini Abeysekara, Harsha Narammalage, Thulangi Siriwardana, Nayana Gunathilaka, Murali Vallipuranathan, Prasanga Serasinghe, Lakmini Liyanage, Nilanka Perera, Guwani Liyanage, Shalindra Ranasinghe, Nilmini Chandrasena

**Affiliations:** Anti Filariasis Campaign, Colombo, Sri Lanka (D. Mataraarachchi, I. Abeysekara, H. Narammalage, M. Vallipuranathan, P. Serasinghe, L. Liyanage); University of Kelaniya, Ragama, Sri Lanka (T. Siriwardana, N. Gunathilaka, N. Chandrasena); Colombo South Teaching Hospital, Colombo (N. Perera, G. Liyanage); University of Sri Jayewardenepura, Nugegoda, Sri Lanka (N. Perera, G. Liyanage, S. Ranasinghe)

**Keywords:** Dirofilaria asiatica, asymptomatic microfilaremia, parasites, zoonoses, vector-borne infections, phylogeny, Sri Lanka

## Abstract

We report 2 cases of asymptomatic *Dirofilaria* microfilaremia in humans in Sri Lanka, detected during night blood surveillance for lymphatic filariasis in 2025. Partial sequence of microfilarial cytochrome c oxidase 1 gene revealed that both infections were caused by the novel species *D. asiatica*, originally described in Hong Kong as *Candidatus *D. honkongensis.

Dirofilariasis is an emerging mosquitoborne zoonosis caused by nematodes of the family Onchocercidae, genus *Dirofilaria *(*1*). Two main species are of relevance in veterinary and clinical practice, *Dirofilaria immitis* (dog heartworm) and* D. repens.* *D. immitis* worms have a wider distribution (Americas, Africa, Europe, and Australasia) and cause potentially life-threatening cardiopulmonary dirofilariasis in canids and felids and pulmonary lesions in accidental human infections. *D. repens* worms are limited to the Old World and cause subcutaneous and ocular dirofilariasis in canines, felines, and in accidental human infections. 

A novel *D. asiatica* nematode species (formerly *Candidatus *Dirofilaria honkongensis) of canine origin was reported in Hong Kong in 2012 and subsequently in other countries in the region; it is gaining prominence as a zoonosis with diverse manifestations including cervical lymphadenopathy, abdominal subcutaneous masses, and subconjunctival nodules (*2*). Stray dogs and putative vector mosquitoes of genera *Aedes*, *Culex*, *Mansonia*, and *Armigeres* are abundant in Sri Lanka. Surveys in Sri Lanka have documented the presence of *D. asiatica* worms among 27.9% of canines with isolation of adult *D. asiatica* worms in subcutaneous nodules, scrota, and spermatic cords (*3*). 

Adult filarial worms and their larval stages were historically identified on the basis of morphology and morphometry (*4*,*5*). However, differentiation of taxa was challenging because of morphologic similarities and the paucity of specific identification features. Using a combined morphologic and molecular approach has enabled differentiation of morphologically similar but genetically distinct species with identification of cryptic species (*2*). Sri Lanka has the highest number of human *Dirofilaria* spp. infections in Asia (*6*).

Most *Dirofilaria* infections in humans are amicrofilaremic (*1*,*5*). The developmental cycle deviates as immature worms localize in aberrant hosts, triggering host inflammatory responses against *Dirofilaria* and *Wolbachia* (essential endosymbiont) antigens that arrest worm development or destroy them, resulting in inflammatory granulomas with indwelling immature worms (*1*). Thus, humans were regarded as dead-end hosts. However, occasional reports of microfilaremic infections have queried this theory (*7*,*8*). We report asymptomatic *D. asiatica* microfilaremia in 2 cases detected incidentally during night blood surveillance for lymphatic filariasis, in Kalutara district, in western Sri Lanka in 2025 (Figure 1). 

**Figure 1:**
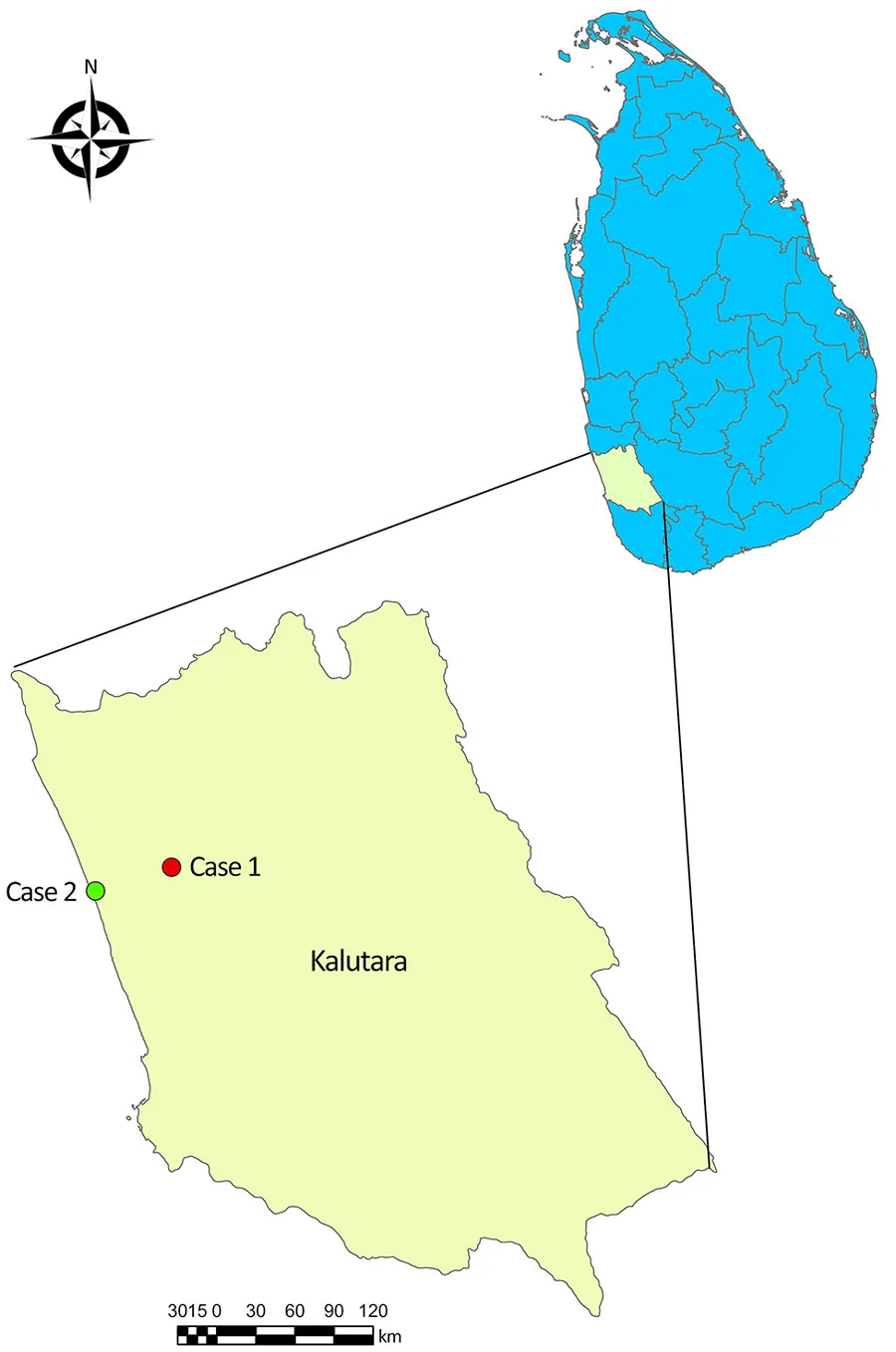
Locations of *Dirofilaria asiatica *microfilaremia cases in study of* Dirofilaria *infection and asymptomatic microfilaremia, Sri Lanka, 2025. Close-up view shows Kalutara district in western Sri Lanka. Cases were detected from night blood smear specimens.

## The Study

Case 1 was in a 60-year-old mason who had a microfilaria count of 7 in a 60-µL night blood smear (NBS) in April 2025. The microfilaria was morphologically compatible with genus *Dirofilaria.* The patient was receiving treatment for diabetes mellitus and hypertension and had longstanding bilateral lower-limb swelling with no identifiable cause and a few palpable inguinal lymph nodes. Other physical examination results were unremarkable. Abdominal ultrasonography and ophthalmology screening results were unremarkable. After obtaining 5 mL of venous blood, we administered diethylcarbamazine (6 mg/kg/d for 12 d) and albendazole (400 mg). His microfilaremia was cleared at 5 weeks posttreatment.

Case 2 was in a 14-year-old boy who had a microfilaria count of 3/60 µL NBS in June 2025. The microfilaria was morphologically compatible with genus *Dirofilaria*. The patient had an occasional dry cough without diurnal variation. Hematology revealed eosinophilia of 568/μL (10.5%). Chest and abdominal imaging and echocardiography were unremarkable. He received doxycycline (100 mg 2×/d for 5 d) and albendazole (400 mg 2×/d for 5 d). Persistence of microfilaremia (1/60µL) at 1 month posttreatment necessitated a repeat course of treatment. His microfilaremia was cleared at 3 months posttreatment.

Giemsa-stained NBSs from both case-patients at 1,000× magnification revealed microfilaria with morphology compatible with *D. repens* and* D. asiatica *worms (*2*). (Table; Figure 2). We performed molecular speciation to establish species identity. We extracted genomic DNA from venous blood of both patients using the QIAGEN Tissue Kit (QIAGEN, https://www.qiagen.com). We amplified mitochondrial cytochrome c oxidase subunit I (COI) gene using the primer pair COIfilF/COIfilR, designed from conserved nematode regions (*9*). We sequenced purified amplicons (QIAquick PCR Purification Kit; QIAGEN) bidirectionally by Sanger sequencing (SeqStudio Genetic Analyzer; Thermo Fisher Scientific, https://www.thermofisher.com) and deposited the resulting sequences in GenBank (accession nos. PZ359659 [AFC615] and PZ359660 [AFC621]). We edited chromatograms manually in BioEdit version 7.2.5 (https://www.updatestar.com) and queried against the National Center for Biotechnology Information nucleotide database using BLASTn (https://blast.ncbi.nlm.nih.gov/Blast.cgi) for homology assessment. We performed multiple sequence alignment using ClustalW in BioEdit. Both isolates were consistently identified as *Dirofilaria asiatica*; BLAST results showed near-complete query coverage (≈100%) and high percentage identity (>99%) to reference *D. asiatica* sequences, confirming species-level identification.

**Table:** Characteristics of *Dirofilaria *microfilariae detected in study of *Dirofilaria asiatica* infection and asymptomatic microfilaremia, compared with those of reference strains, Sri Lanka, 2025*

Morphology	Microfilaria in this study	*D. repens *microfilaria†	*D. asiatica *microfilaria‡
Sheath	Absent	Absent	Absent
Mean total body length, µm	349	369 +10	300.3
Mean width, µm	8	8.87+0.5	5.7
Cephalic space			
Mean length, µm	2.5	2.96 + 1.18	3.27
Location	Round anterior end	Round anterior end	Not specified
No. nuclei	2–3	2 nuclei	2–3 nuclei
Tail	Tapering, curved, free of nuclei	Tapering, curved, free of nuclei	Tapering, curved, free of nuclei

*Specimens in this study were human blood smears stained with 10% Giemsa; we observed a mean of 10 microfilariae per specimen. Characteristics were obtained using Olympus microscope (original magnification ×1,000) and cell analysis software ScopeImage version 9.0 (https://www.bioimager.com). †Magnis et al. (*5*). ‡Colella et al. (*2*) Specimens were stained with 0.1% methylene blue.

**Figure 2:**
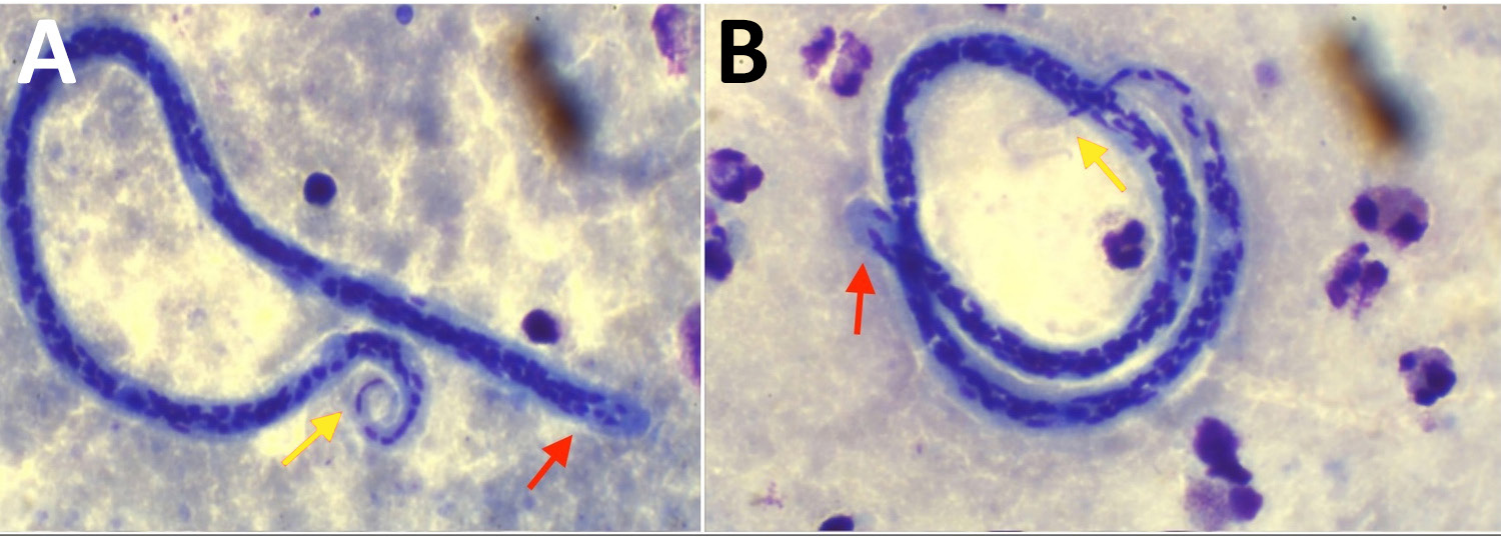
Microfilaria detected in Giemsa-stained thick blood smears from case-patients 1 (A) and 2 (B) in study of* Dirofilaria *infection and asymptomatic microfilaremia, Sri Lanka, 2025. Red arrows indicate the anterior end (cephalic space) with 2–3 distinct nuclei; yellow arrows indicate the tails free of any nuclei. Original magnification ×1,000.

We constructed a maximum-likelihood phylogenetic tree in MEGA version 11 (*10*), using the best-fit substitution model selected by Bayesian Information Criterion (Figure 3). We assessed branch support using 1,000 bootstrap replicates and genetic distances estimated under the Kimura 2-parameter model. The resulting tree resolved 6 well-defined major clades, corresponding to *D. asiatica*, *D. repens*, *D. immitis*, *D. ursi*, *Onchocerca* sp., and *Onchocercidae* sp.; the deepest nodes separating the clades were supported at 10%–23%. Both study isolates, AFC615 and AFC621, clustered firmly within the *D. asiatica* clade alongside reference sequences also obtained from GenBank (accession nos. PV523839, PV523835, PV523843, and PV523842). Isolate AFC615 grouped closely with PV523839 and PV523835; bootstrap support was 99% at the terminal node and 97% and 91% at the supporting internal nodes. Isolate AFC621 formed a pair with reference sequence PV523842; the pair nested within the broader *D. asiatica* clade at 43% support. The clustering we observed was fully consistent with the BLASTn results, confirming both isolates as genuine *D. asiatica*.

**Figure 3:**
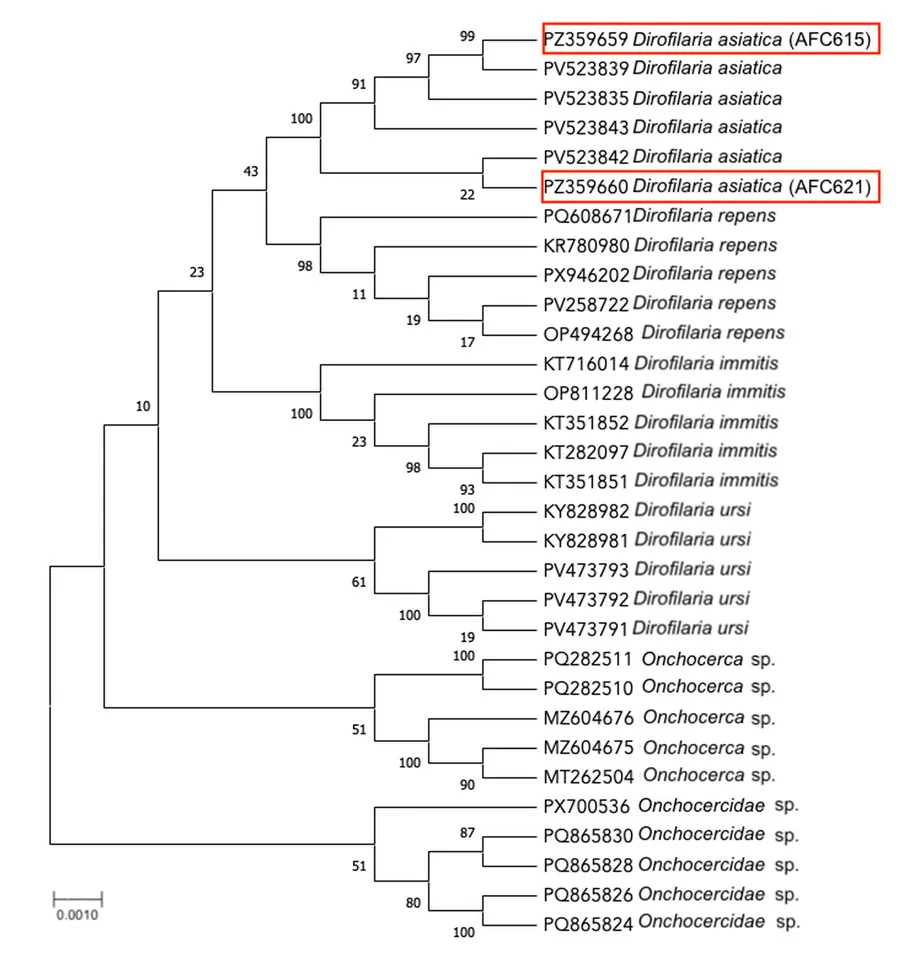
Maximum-likelihood phylogenetic tree generated in study of *Dirofilaria asiatica* infection and asymptomatic microfilaremia, Sri Lanka, 2025. Tree includes partial mitochondrial *cox1* gene sequences from 2 *D. asiatica* isolates in this study (red boxes) and curated reference sequences representing *D. asiatica*, *D. repens*, *D. immitis*, and *D. ursi*; we included *Onchocerca* sp. and *Onchocercidae* sp. as non-*Dirofilaria* outgroups. We constructed the tree in MEGA version 11 (www.megasoftware.net) under the Kimura 2-parameter substitution model, selected as the best-fit model by Bayesian Information Criterion. Node support was assessed using 1,000 bootstrap replicates; numbers at nodes indicate bootstrap values >50%. GenBank accession numbers are provided. Scale bar represents nucleotide substitutions per site.

Overall, the phylogenetic tree demonstrated clear separation of the 6 major clades (*D. asiatica*, *D. repens*, *D. immitis*, *D. ursi*, *Onchocerca* sp., and *Onchocercidae* sp.), with both study isolates aligning unambiguously within the *D. asiatica* lineage. The range of bootstrap support observed within this clade (22%–99%) reflects a degree of intraspecific genetic variation among *D. asiatica* sequences from different geographic sources, while clearly resolving both Sri Lanka isolates as *D. asiatica* (Figure 3).

Human *Dirofilaria* infections have been reported in Sri Lanka over many decades (*6*), although not specifically microfilaremia caused by *D. asiatica* filarial worms. Case-patient 1, who had underlying conditions, was probably immunodeficient to some extent, enabling the maturation of *D. asiatica* larvae into adults, whereas case-patient 2 may have had multiple exposure events. 

A literature review revealed use of 3 treatment regimens with no preference of one over the other (*11*). The first 2 regimens were diethylcarbamazine/albendazole combination therapy or doxycycline/albendazole combination therapy targeting the endosymbiont *Wolbachia*. The third regimen was ivermectin (200 μg/kg/d for 4 d) and doxycycline (100 mg 2×/d for 7 d) (*12*). We used the diethylcarbamazine/albendazole and doxycycline/albendazole regimens because ivermectin was not registered for human use in Sri Lanka at the time of treatment. Although both treatment regimens achieved clearance of microfilaremia, the viability of adult worms remains uncertain; attempts to locate adult worms were unsuccessful. Thus, we deliberated recommendations for annual follow-up by blood screening for 5 years.

## Conclusions

Our report establishes the presence of accidental *D. asiatica* infections among humans in Sri Lanka who were asymptomatic with microfilaremia. Further investigations will support the establishment of dirofilariasis as a zoonotic public health problem. We recommend routine administration of antifilarial drugs to dogs and cats, control of mosquitoes, and raising awareness of dirofilariasis. 
